# Food allergy and anaphylaxis – 2046. Cloning and characterization of the gene for acidic thaumatin-like protein, an important allergen from sapodilla plum (*manilkara zapota*)

**DOI:** 10.1186/1939-4551-6-S1-P131

**Published:** 2013-04-23

**Authors:** YP Venkatesh, HG Ashok Kumar, VL Hegde, SM Shetty

**Affiliations:** 1 Biochemistry & Nutrition, Csir-Central Food Technological Research Institute, Mysore, India

## Background

Allergic reactions to sapodilla ingestion are rare; a 21 kD protein was recognized as an allergen in sapodilla extracts by IgE-immunoblot [1]. Further, the allergen was identified as a basic thaumatin-like protein (TLP) by its N-terminal sequence (ATFDIQNNC-) and isoelectric point. The major purpose of this study was to identify additional allergens from this tropical fruit.

## Methods

A case of oral allergy syndrome to sapodilla and custard apple was investigated following approval by Institutional Ethics Committee. Sapodilla allergy was confirmed by diagnostic tests (SPT and allergen-specific IgE). Sapodilla proteins were separated on SP-Sepharose by adsorption at pH 4 followed by step elution at pH 5 (SP1), and with increasing NaCl – 0.1 M (SP2) and 0.2 M (SP3). Forward primers and nested reverse primers specific to the SP1 component were designed based on its N-terminal sequence and conserved regions of homologous plant TLPs. PCR was performed using sapodilla (cv. cricket ball) leaf genomic DNA as template.

## Results

SPT and allergen-specific IgE were positive. ELISA revealed that IgE from allergic serum recognized two 21 kD proteins – one in the SP1 pool and the other in the SP2 pool; the 21 kD protein in SP2 was identified as basic TLP. The N-terminal sequence of SP1 component was found to be ATFDVVNQCTFTVWAGASPGGGKQL- which was identified as an additional TLP. Sequence analysis of overlapping PCR products revealed an almost full-length gene (603 bases; GenBank accession JN624813.1) corresponding to acidic TLP (residues 8–207 of sapodilla acidic TLP and lacking the N-terminal 7 residues). Phylogenetic analysis shows that sapodilla acidic TLP is evolutionarily related to the allergenic TLPs from olive and kiwi fruits, all belonging to the order Ericales.

## Conclusions

A partial gene coding for sapodilla acidic TLP representing 97% of the mature sequence has been cloned. Sapodilla acidic TLP has weak β-1,3-glucanase activity and is an important allergen belonging to the TLP family of pollen and fruit allergens [2].
